# Influence of Natural Honey on Biochemical and Hematological Variables in AIDS: A case study

**DOI:** 10.1100/tsw.2006.331

**Published:** 2006-02-02

**Authors:** Noori S. Al-Waili, Thia N. Al-Waili, Ali N. Al-Waili, Khelod S. Saloom

**Affiliations:** Al-Waili's Charitable Foundation for Research and Trading, New York, USA, and Dubai Specialized Medical Center, Islamic Establishment for Education, UAE

**Keywords:** AIDS, honey, nitric oxide, prostaglandin, blood indices, blood biochemistry, United States

## Abstract

Honey lowers prostaglandins and elevates nitric oxide (NO) in various biological fluids in normal persons. NO and prostaglandin play a role in pathogenesis of AIDS. The study was designed to assess the effect of natural honey on prostaglandins and NO levels, blood indices and biochemical tests in a 40 year-old woman with AIDS. This presentation is a case story of a 40 year-old women with a long history of AIDS treated with 80g of natural honey. Plasma and urinary prostaglandin F2 alpha and thromboxane B2 levels, plasma, urine and saliva content of NO-end product (total nitrite) and hematological tests were estimated before and 3 hours after oral consumption of 80g of natural honey. These variables, in addition to biochemical tests, were re-estimated after 21 days of daily consumption of 80g of natural honey. Results showed that prostaglandins level compared with normal subjects were elevated in patient with AIDS. Natural honey decreased prostaglandins levels, and elevated NO-end product, percentage of lymphocytes, platelet count, and serum protein, albumin and copper levels. It might be concluded that natural honey decreased prostaglandins level, elevated NO production and improved hematological and biochemical tests in a patient with a long history of AIDS.

## INTRODUCTION

Many reports have shown that honey has wide biological and therapeutic effects. It has been used for a long time to treat wounds and burns. It was mentioned in the Holy Quran as a cure for all illnesses (And thy LORD taught the bee to build its cells in hills, on tree and in men's habitations, then to eat of all the produce of the earth and find with skill the spacious paths of its LORD, there issues from within their bodies a drink of varying colors, wherein is healing for men, verily in this is a sign for those who give thought. Surat Al-Nahl (The Bees), Aya 69). Honey has been used for treatment of respiratory diseases, urinary diseases, gastrointestinal diseases, and skin diseases including ulcers, wounds, eczema, psoriasis and dandruff[1,2,3]. It has an excellent antibacterial activity against a wide range of human pathogens[4,5]. Recently, we have found that topical honey application is useful for treatment of recurrent herpetic lesions[6]. Honey stimulated proliferation of B and T lymphocytes in cell culture and stimulated monocytes to release cytokines, which activated immune responses[7]. Recently we have found that various honeys contained different amount of nitric oxide (NO) metabolites and intravenous honey could increase urinary nitrite excretion in animals[8]. Oral honey ingestion increases plasma, saliva and urinary contents of NO metabolites[9]. In normal subjects, we found that honey could reduce plasma prostaglandin E2, prostaglandin F2 alpha and thromboxane B2 levels[10]. Urinary excretion of prostaglandins was reduced by honey[11]. In addition, two weeks consumption of honey by normal subjects increased concentration of antioxidants in the blood and slightly increased the percentage of lymphocytes, hemoglobin, red blood cell and platelet count[12]. A 33% increase in the serum copper level was obtained[12].

The objective of this study was to find whether raw unprocessed honey could affect prostaglandin and NO production in a patient with a long history of AIDS.

## CASE REPORT

A 40 year-old woman suffering from AIDS for 10 years was studied. She had HIV-1 infection since 1992. At time of entry, she complained of tiredness and anxiety. No other symptoms were found on systemic review. Physical examination revealed normal findings. The patient did not receive any antiviral therapy during the six months preceding the test. Hematological and biochemical investigations were performed after 16 hours of fasting. Plasma and urinary PGF2 alpha and thromboxane B2 were estimated using the quantitative analysis performed with use of an enzyme linked immunosorbant assay (ELISA, Neogen Corporation, USA). Total nitrite (stable NO end product) was estimated in plasma, urine and saliva using Griess Reagent (Assay Designs, USA). Prostaglandins and total nitrite were estimated after 16 hours of fasting. Then the patient was asked to intake 80g of natural honey (Multifloral). After 3 hours, estimation of blood indices, prostaglandins and total nitrite was repeated. The patient was asked to intake 80g of natural honey once daily for a maximum of 21 days. On day 22, total nitrite was estimated in the urine, saliva and plasma samples and prostaglandins were estimated in plasma and urine samples. Hematological and biochemical tests were repeated on day 22 after honey intake. Results showed that total nitrite was increased in plasma and saliva after 3 hours and after 21 days (Table 1). The urinary nitrite level was increased from 30.7µmol/L before honey intake to 56µmol/L after 21 days of honey intake. Prostaglandin F2 alpha and thromoboxane B2 levels were decreased after 3 hours and after 21 days in plasma and urine samples. Platelet count and percentage of lymphocytes were increased 3 hours after honey intake and after 21 days (Table 1). Biochemical tests repeated after 21 days of treatment showed that there was an increase in serum protein from 6.8g/dl to 7.2g/dl, serum albumin from 4g/dl to 4.5g/dl, fasting blood sugar from 74g/dl to 85g/dl, serum copper level from 97mg/dl to 120mg/dl, uric acid from 2.3mg/dl to 2.7mg/dl and alkaline phosphates from 62U/L to 94U/L. Gamma GT was decreased from 26U/L to 12U/L and SGPT from 12U/L to 9U/L. It seems that natural honey could increase total nitrite level in the urine, plasma and saliva and decrease prostaglandins concentration in the urine and plasma. Honey also increased percentage of lymphocytes, serum protein, serum albumin and serum copper level.

## DISCUSSION

The results showed that prostaglandins levels were elevated in a patient with AIDS. Natural honey reduced elevated prostaglandins levels and increased NO production, serum protein and albumin, platelet count, and percentage of lymphocytes. These changes might help patients with AIDS and ameliorate their health problems. Honey increases NO and reduces prostaglandins in various biological fluids[8-10]. It has been demonstrated that NO has an antiviral effect and there is some evidences of NO-activity decreasing or blocking HIV-1 replication[13]. NO-donors inactivate the HIV-1 encoded protease and reverse transcriptase[14]. NO is a very important mediator of immune responses[15]. Sera from person

**Table 1.**
**Effect on honey consumption (80 g/day) on nitric oxide-end product (nitrite), prostaglandins and blood indices in the patient with AIDS.**

| Variables | Time after consumption | | |
|---|---|---|---|
| | O | 3 h | 22 days |
| Prostaglandin F2 alpha (plasma) ng /m1 | 1.30 | 0.23 | 0.12 |
| Prostaglandin F2 alpha (urine) ng/m1 | 2.25 | 0.99 | 2.2 |
| Thromboxane B2 (plasma) ng /m1 | 0.65 | 0.3 | 0.2 |
| Thromboxane B2 (urine) ng /ml | 0.30 | 0.10 | 0.2 |
| Total Nitrite (plasma) μmo1/L | 18.9 | 34.82 | 73 |
| Total Nitrite (saliva) μmo1 /L | 50.3 | 139.5 | 117 |
| White blood cell Count K/uL | 4.7 | 4.69 | 4.5 |
| Lymphocyte (%) | 39 | 55 | 50 |
| Granulocyte (%) | 55 | 36 | 45 |
| Monocyte (%) | 5 | 7 | 5 |
| Eosinophil (%) | 1 | 2 | 1 |
| Hemoglobin (g/dl) | 11.8 | 11.8 | 11.9 |
| Platelet K/uL | 133 | 267 | 277 |

with AIDS contain inhibitors of lymphocytes proliferation such as prostaglandin[16]. PGE2 produced by HIV-infected antigen presenting cells causes lymphocytes apoptosis[17]. Cyclooxygenase inhibitors blunted this effect[17]. In a murine model of AIDS, mice treated with prostaglandin inhibitor showed higher total leukocytes and platelet and higher helper cells[18]. Plasma prostaglandin F2 alpha and thromboxane B2 levels were found to be higher in the patient with AIDS as compared with normal subjects[10]. Natural honey decreased elevated plasma prostaglandin F2 alpha and thromboxane B2 to, or even below, the mean level of prostaglandin found in normal subjects[10]. In addition, natural honey decreased urinary prostaglandins concentration in a patient with AIDS. Plasma and saliva levels of total nitrite in the patient with AIDS were lower than those in normal subjects[9]. Natural honey increased NO end product in saliva, plasma and urine samples. However, high output of NO production may represent a potential risk to the human[15]. Nevertheless, honey did not provoke great increments in NO-end products. Twenty-three years ago, we had found that prostaglandin was a potent immunosuppressive and accordingly it had been suggested that inhibition of prostaglandin might increase immunity in conditions associated with the over production of prostaglandins such as chronic inflammations and malignancies[19]. Moreover, we have found that honey increased antibody production against thymus dependent and thymus independent antigens[20]. These honey's effects may help patients with AIDS.

Many Phase II, double-blind, placebo-controlled studies conducted on patients with AIDS showed various effects of placebo on the patient's health and parameters[21-25]. However, there has been vigorous debate over the use of placebo controls in clinical trials in human subjects where active treatments are already in widespread use, particularly the use of placebo controls in trials of products for AIDS[26]. In addition, there is ethical consideration for using placebo in certain conditions. In our case study, it was difficult to prepare placebo similar to honey. Furthermore, the patient knew that she was using honey as a nutrient. Nevertheless, the patient's care and handling from the medical staff might affect her condition. We suggest that further investigations, recruiting larger numbers of patients who consume natural honey for a longer period and who are compared with a control group using artificial honey (fructose and glucose), might substantiate the result of this observation.

## ACKNOWLEDGEMENTS

The authors would like to thank Haj Saeed Lootah, Chairman of Trustee, Islamic Establishment for Education, Dubai, for his kind support. Mr Nader S. Boni provided assistance.

## BIOSKETCH

**Noori S Al-Waili, MD, DOG, PhD, CHT,** Director of Clinical Research, Life Support technologies INC, Chronic Wound Treatment and Hyperbaric Medicine Center, The Mount Vernon Hospital, Mount Vernon, New York, United States of America. Email: Dr AL-Waili is a physician and research scientist who has gained both in-depth clinical and extensive research experiences in his medical career (20 years). He has been publishing more than 160 scientific publications in nephrology, urology, hypertension, diabetes, malignancy, immunity, alternative medicine and biomedical sciences, including wide range of new therapeutic measures. He has many theories and discoveries in modern and alternative medicine, which have been published in top journals in USA, UK, Australia, Europe and others. He has many exiting publications in nephrology/urology including peritoneal macrophages transfusion, enuresis, renal colic, frequency of micturiton, and others. At time, his main interest is focused on nephrology/urology, wound care and hyperbaric medicine as well as alternative medicine. He is working as a member of Editorial Board of two medical journals, a member of evaluator panel, Current Drug, UK, and a reviewer for many medical and biomedical journals.
